# Proximal 21q deletion as a result of a *de novo* unbalanced t(12;21) translocation in a patient with dysmorphic features, hepatomegaly, thick myocardium and delayed psychomotor development

**DOI:** 10.1186/s13039-016-0220-5

**Published:** 2016-02-04

**Authors:** Cathrine Jespersgaard, Ida N. Damgaard, Nanna Cornelius, Iben Bache, Niels Knabe, Maria J. Miranda, Zeynep Tümer

**Affiliations:** Department of Clinical Genetics, Applied Human Molecular Genetics, Kennedy Center, Copenhagen University Hospital Rigshospitalet, Glostrup, Denmark; Department of Paediatrics, Copenhagen University Hospital, Herlev, Denmark; Wilhelm Johannsen Centre for Functional Genome Research, Department of Cellular and Molecular Medicine, University of Copenhagen, Copenhagen, Denmark

**Keywords:** Partial monosomy, Monosomy 21, Translocation, 21q22

## Abstract

**Background:**

IInterstitial 21q deletions can cause a wide spectrum of symptoms depending on the size and the location of the deletion. It has previously been suggested that the long arm of chromosome 21 can be divided into three regions based on the clinical severity of the patients and deletion of the region from 32.3 Mb to 37.1 Mb was more crucial than the deletion of other regions.

**Case Presentation:**

In this study we describe a female patient with dysmorphic features, hepatomegaly, thick myocardium and psychomotor delay. Conventional karyotyping was initially interpreted as full monosomy 21, but subsequent chromosome microarray analysis suggested an approximately 18 Mb partial monosomy. Re-evaluation of the karyotype and fluorescence in situ hybridization revealed deletion of the proximal 21q11.2-q22.11 segment and insertion of 21q22.11-qter to 12qter. The deletion of the present case overlaps with two of the proposed regions including part of the proposed crucial region.

**Conclusions:**

This report emphasizes the relevance of investigating suspected full monosomies with high resolution methods and FISH in order to investigate the extent of the deletion and the presence of more complex rearrangements.

## Background

Full monosomy of chromosome 21 is a rare finding but its real frequency is unknown, as some of the reported cases, which were analysed with G-banded chromosomes, were subsequently shown to be partial monosomies when investigated with fluorescence in situ hybridization (FISH) or other molecular techniques [1]. Full monosomy 21 has thus only been reported and confirmed in 14 cases [2–16]. Burgess et al. have suggested that full monosomy 21 cases should be investigated for cryptic unbalanced rearrangements and chromosomal mosaicism as true monosomies may not be viable in most cases [17]. Partial monosomy on the other hand has been reported in more cases, but it is still a rare finding and the patients present with a broad spectrum of phenotypes partly correlated with the size and localization of the deletion.

In this study, we report a chromosome rearrangement where the proximal 21q11.2-q22.11 segment was deleted and 21q22.11-qter was inserted to 12qter. The clinical features of the patient, who was referred to genetic diagnosis at age 1 week, are described in comparison with those of other reported cases with overlapping deletions.

### Case Presentation

The patient was a female delivered by acute Caesarean section at gestational age of 38 + 1 weeks. Labour was medically induced because of the large size of the foetus and shifted to Caesarean section due to imminent asphyxia. After birth the pH of the umbilical cord blood was 7,16. Apgar scores were 3/1 and 7/5. Birth weight was 4618 grams and birth height was 49 cm.

Due to respiratory distress she was treated with NCPAP (nasal continuous positive airway pressure) for a total of 13 days. She appeared “puffy” and with thick subcutaneous tissue resembling diabetic foetopathy, but with normal circulation. The mother was tested for gestational diabetes twice during the pregnancy with normal results. Face of the newborn was flushing and asymmetrical with prominence of left cheek and chin. A subtle torticollis twisting toward the left side was noticed. A sagittal swelling was present in the forehead. She had small and low-set ears, and the right one was crumpled. On the right hand the 3rd finger was overriding the 2nd. The left foot was inwardly rotated but redressable. At birth, she had sinistra convex position, probably due to the intrauterine posture. X-ray of thorax revealed cardiomegaly, confirmed by echocardiography, which also revealed cardiac myopathy with atrial septal defect. Abdominal ultrasound revealed hepatomegaly. She was hypotonic and had decreased motor activity. Eye examination was normal. MRI of cerebrum revealed hypomyelination. Hip abduction was restricted and ultrasonography demonstrated bilateral hip dysplasia successfully treated with Dennis-Browne brace. Metabolic screening was normal. Initially she had problems with sucking and feeding was supplemented by naso-gastric tube and bottle. She was discharged from the hospital when she was about 1-month-old and followed closely by a team of paediatric specialists and regular physiotherapy.

**Fig. 1:**
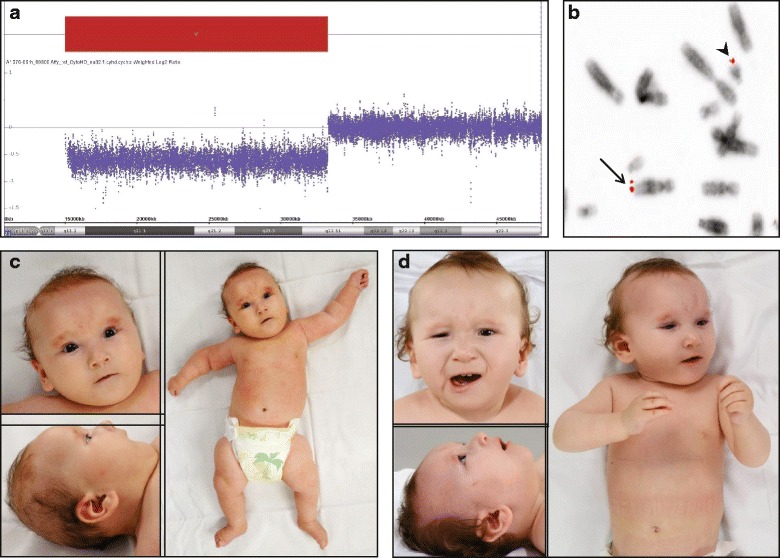
Chromosomal microarray overview from chromosome 21 displaying the deletion at 21q11.2q22.11 (15218106-33265774)x1 (**a**); FISH signals on metaphase chromosomes of the patient using the 21qter probe. One of the signals is on the normal chromosome 21 (arrow head) and the other signal on the derivative chromosome 12 (arrow) (**b**); The clinical pictures of the patient at age 5 months (**c**) and at age 1 year (**d**)

Clinical examination at age 1 year showed dysmorphic facial features including small eyes, low-set ears, and asymmetrical chin with a deviation of the lower jaw towards the left (Fig. 1c). She had levoscoliosis and synchondrosis of the left elbow was suspected. Her psychomotor development was delayed. She was able to sit but she was still hypotonic and had tendency to use the left extremities more. Her fine motor functions improved gradually. She started to walk at the age of 22 months. Her scoliosis became less pronounced. Repeated eye examinations revealed slightly impaired vision of the right eye. She did not have eating problems at this age (22 months). Language development was slightly delayed for the age (22 months), as she could only make sounds without proper words. She started to use sign language and was affiliated to a special day care.

The patient is the second child of healthy but consanguineous parents (the father is second nephew to the mother). The 3-years-older sister was reported healthy although she was a late walker.

## Materials and methods

Cytogenetic analysis was carried out using Giemsa banded metaphase chromosomes prepared from peripheral blood lymphocytes. Genomic DNA was prepared from whole blood using standard procedures. Chromosome microarray was carried out using CytoScan HD array and data were analysed using ChAS software (Affymetrix, CA, USA). FISH-analyses (Fluorescence *in situ* hybridization) were carried out on metaphase chromosomes with commercial subtelomeric probes for 21qter, 12pter and 12qter (Vysis Inc., Abbott Laboratories SA, IL, USA) according to manufacturer’s recommendations. STR (short tandem repeat) markers on chromosome 21 was analysed with the Elucigene QSTR*R-21 kit as recommended by the manufacturer (Elucigene, Manchester, United Kingdom).

## Results and discussion

The initial cytogenetic analysis of the patient, carried out by G-banded chromosomes, suggested monosomy 21, with karyotype 45,XX,-21. Chromosomal microarray analysis identified an approximately 18 Mb deletion at 21q11.2-q22.11 (chr21:15,218,106-33,265,774) (UCSC Genome Browser, http://genome.ucsc.edu/, February 2009GRCh/hg19 release) (Fig. 1a). The initial karyotyping was re-evaluated revealing that the terminal 21q was inserted to the 12qter. This was confirmed with FISH analyses using subtelomeric probes for 21qter, 12pter and 12qter. The patient’s karyotype was hence revised as 45,XX,der(12)t(12;21)(pter- > q24.33::q22.11- > qter). arr[hg19] 21q11.2q22.11 (15,218,106-33,265,774)x1. Chromosomes of the parents were investigated by cytogenetic analysis and FISH using the subtelomeric probes for 21q and 12q and both had normal karyotypes. STR analyses showed that the deletion had occurred on the paternal allele (data not shown).

**Fig. 2:**
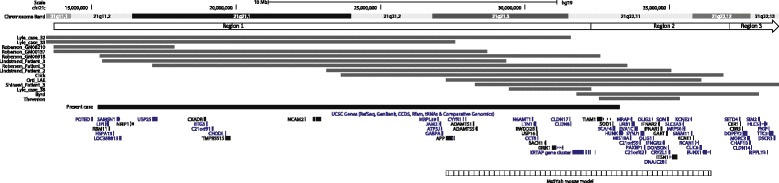
The genomic region deleted in the present patient (black horizontal bar) and in the previously reported cases (grey bars) [21, 23–29, 33]. The Ms5Yah mouse model (horizontal striped bar) and the regions (Region 1–3, horizontal white bars) proposed by Lyle et al. [21] are also shown. The genes shown in the figure are UCSC genes (genes that have a corresponding entry in the Protein Data Bank or the transcript has been reviewed or validated by either the RefSeq, SwissProt or CCDS staff)

Comparison of the phenotypes of the reported partial monosomy 21 patients is difficult as in many cases the monosomy is not pure or patients carry other rearrangements including translocations, deletions or duplications involving other chromosomes [18–22]. In the literature there are more than 30 pure partial monosomy 21 cases where the deletion breakpoints are investigated with high resolution methods [21, 23–36]. Only 14 of these patients have deletions overlapping with the deletion of the present case (Fig. 2 and Table 1) [21, 23–29, 33]. The common features of these patients include developmental delay, short stature, low birth weight, microcephaly, dysmorphic features, neonatal seizures, clinodactyly, cardiac anomalies. The overlapping symptoms of the present case and the previous reported cases are developmental delay, low set ears, scoliosis and cardiac anomalies. The patient reported by Roberson et al (GM06918) has a deletion similar to that of the present case [23]. The overlapping features of these two patients are skeletal abnormalities, dysmorphic features and developmental delay similar to the common features for all the patients with partial monosomy in this region.

**Table 1:** Summary of the clinical phenotypes of the patients presented in Fig. 2

Clinical features	Present case	Lyle case 32	Lyle case 33	Roberson GM08210	Roberson GM00137	Roberson GM06918	Lindstrand Pt3	Roberson Pt3	Lindstrand Pt2	Click	Orti LAE	Shinawi Pt3	Lyle case 38	Byrd	Thevenon
Sex	F	U	U	F	M	M	M	F	F	F	U	F	U	F	M
Age at latest examination^a^	2	U	U	U	U	U	6	6	0.8	0.2	U	U	U	5	6
Development															
Intellectual disability	+	+	+	+	+	+	+	+	+			+			
Hearing loss										+					
Short stature		+				+									
Low birth weight							+		+	+				+	+
Delayed or no language	+						+								+
Feeding difficulties									+						
Neurological															
Hypotonia	+	+													+
Hypertonia		+													
Craniofacial features															
Facial asymmetry	+			+											
Microcephaly		+													
Low anterior or posterior hairline		+				+		+		+					+
Frontal bossing				+								+			+
Synophrys										+					
Low set ears	+								+	+					+
Large ears		+								+					
Bulbous nose tip										+					
Broad or depressed nasal bridge		+		+						+		+			+
High or cleft palate		+		+		+								+	
Broad mouth		+													
Micrognathia						+									
Downward slanting palpebral fissures						+			+	+				+	+
Strabismus								+		+		+		+	+
Small eyes	+											+			
Hypertelorism									+			+			
Amblyopia								+							
Epicanthal folds												+		+	
Other															
Gastroesophageal reflux								+	+						
Congenital heart defect	+						+		+	+				+	+
Hepatomegaly	+														
Scoliosis	+									+					
Distal limbs abnormalities	+			+			+							+	
Clinodactoly of the fifth finger		+													
Palmar crease		+												+	+

*M* male, *F* female, *U* unknown; ^a^in years

Our patient has a deletion that spans more than 60 Refseq genes including a *KRTAP* gene cluster comprising 16 genes (Fig. 2) and it is difficult to predict the contribution of these genes to the phenotype. Based on a comparison of the previously reported patients (n = 11) with partial monosomy 21, Lyle et al divided 21q into three regions [21, 23] and the present deletion spans Region 1 and approximately 1 Mb of Region 2. The approximately 32.3 Mb region (Region 1) from the centromere to 21q11.2-q22.11 contains more than 60 genes and the patients with deletions within this region tend to have a severe phenotype. Region 2 (32.3 – 37.1 Mb, 21q22.11-q22.12) contains more than 30 genes and none of the 11 patients had a deletion spanning the entire region, suggesting that this region could contain genes, codeletion of which are not tolerated [21]. The distal Region 3 (~37.1 – 38.6 Mb to 21qter, 21q22.12-q22.3) harbouring more than 130 genes, causes a milder phenotype in monosomic state. Patients with Region 1 and/or Region 2 deletions may present with more severe phenotypes compared to patients with deletions of Region 3 [21, 23]. In the literature there are two patients with deletions spanning Region 2 [27, 28]. The patient reported by Shinawi et al. was mosaic, where the deletion encompassing Region 2 was observed in 15 % of the cells, while the other cells had a smaller deletion distal to Region 2 [27]. This is in line with Lyle’s hypothesis suggesting that codeletions of two or more genes of this region are not tolerated. A mouse model of monosomy 21 with an approximately 9 Mb deletion corresponding to the human *APP*-*RUNX1* region (distal part of Region 1 and whole Region 2) shows developmental delay, size and weight reduction, thrombocytopenia, motor coordination deficiencies , and spatial learning and memory impairments (Fig. 2) [37]. Notably, the deletion of the region influences the viability as the transmission of the allele with the deletion is reduced, supporting Lyle’s hypothesis. However, the patient reported by Byrd et al. does not fit this hypothesis and description of further patients with partial monosomy 21 is necessary to clarify the importance of Region 2 and hence the dosage effect of the genes within this region.

## Conclusion

This report emphasizes the relevance of investigating suspected full monosomies with high resolution methods and FISH in order to investigate the extent of the deletion and the presence of more complex rearrangements.

### Consent

Written informed consent was obtained from the patient for publication of this Case Report and any accompanying images. A copy of the written consent is available for review by the Editor-in-Chief of this journal.

## Abbreviations

APP: Amyloid beta A4 protein
ChAS: chromosome analysis suite
FISH: Fluorescence in situ hybridization
G-banding: Giemsa banding
KRTAP: Homo sapiens keratin associated protein
Mb: Mega bases
NCPAP: nasal continuous positive airway pressure
RUNX1: Runt-related transcription factor 1
